# Vertebral fractures in patients with inflammatory bowel disease COMPARED with a healthy population: a prospective case-control study

**DOI:** 10.1186/1471-230X-12-47

**Published:** 2012-05-14

**Authors:** Ma Angeles Vázquez, Enrique Lopez, Ma José Montoya, Mercè Giner, Ramón Pérez-Temprano, Ramón Pérez-Cano

**Affiliations:** 1 Medicine Department, University of Seville, Seville, Spain; 2 Osteoporosis Unit, University Hospital “Virgen Macarena”, Sevilla, Spain; 3 Internal Medicine Service, Hospital Juan Ramón Jiménez, Huelva, Spain; 4 Osteoporosis Unit, University Hospital “Virgen Macarena”, Av. Dr. Fedriani s/n, 41009, Seville, Spain

**Keywords:** Morphometric vertebral fractures, Inflammatory bowel disease, Bone mineral density, Bone remodeling

## Abstract

**Background:**

A prospective study was performed to compare the prevalence of morphometric vertebral fractures (MVF) between patients with inflammatory bowel disease (IBD) and healthy subjects and to identify predictive factors of fracture.

**Methods:**

A total of 107 patients with IBD (53 with Crohn’s disease and 54 with ulcerative colitis) and 51 healthy subjects participated in the study. Information about anthropometric parameters, toxins, previous fractures, and parameters related to this disease were evaluated. The index of vertebral deformity, bone mass density (BMD), and biochemical parameters were calculated.

**Results:**

A total of 72 fractures were detected in 38.32% of patients with IBD, and 10 fractures were detected in 13.73% of healthy subjects; the risk of fracture in patients with IBD was higher than that in control subjects (OR, 4.03; 95% CI, 1.652–9.847; *p* < 0.002). We found no correlation between fracture and BMD in patients with IBD (lumbar spine, r = −0.103, *p* = 0.17 and femoral neck, r = −0.138, *p* = 0.07). Corticosteroid treatment was not associated with prevalent vertebral fractures nor with taking corticosteroids (r = 0.135, *p* = 0.14) or the duration for which they were taken (r = 0.08, *p* = 0.38), whereas this relationship was present in the controls (r = −0.365, *p* = 0.01). In the multivariate analysis, none of the measured parameters were significantly predictive of fracture, only to manifested IBD. Hypovitaminosis D was observed in 55.14% of patients with IBD.

**Conclusions:**

The prevalence of morphometric vertebral fractures is higher in patients with IBD than in the healthy population, without association with BMD or corticoid treatment. Simply having IBD was proven to be a predictive factor of fracture. We observed a high incidence of hypovitaminosis D in patients with IBD.

## Background

Inflammatory bowel disease (IBD) has been associated with low bone mass and a high prevalence of osteopenia and densitometric osteoporosis [1-4]. The etiopathogenesis and risk factors leading to this complication remain controversial [5-7]. An increased risk of developing an osteoporotic fracture because of low bone mass is predictable.

Osteoporosis is associated with a high risk of spinal fracture. Clinical problems of vertebral fractures include severe and chronic back pain, height loss, spinal deformity, and disability. New vertebral fractures are associated with substantial increases in back pain and increasing functional limitations, even those that are not recognized clinically, and are predictive of additional fractures. Vertebral fractures may represent a serious lifelong handicap among younger patients [8].

Data regarding the prevalence and risk of fracture in patients with IBD are currently scarce; thus, clinical relevance in the context of this disease remains to be clarified.

Studies assessing the risk of fracture in IBD are scarce and controversial and use different methodologies [8-12], and those analyzing possible risk factors associated with the appearance of these fractures are much more limited. Most are focused on patients with Crohn’s disease (CD) [8-11] and evaluate the prevalence of fractures in patients with low bone mass, which introduces an important bias [8]. Other studies evaluate the prevalence of vertebral crush fractures only in the IBD group without quantifying these fractures in a control group with similar characteristics [8-12].

We found no publications studying the prevalence of morphometric vertebral crush fractures in patients with IBD who were consecutively included during 1 year in a specialized unit and compared with a control group of subjects with similar ages and anthropometric characteristics during the same period of time. Thus, we undertook this experiment to prove that there is a higher prevalence of fractures in patients with IBD than in healthy subjects.

The primary aim of the study was to evaluate the prevalence of morphometric vertebral crush fractures in patients with IBD and to compare it with the prevalence in a control population with similar characteristics. As a secondary goal, we aimed to identify differences in biochemical parameters and bone mineral density (BMD) between the two groups to detect possible predictive factors of fracture in patients with IBD.

## Methods

### Patients

This was a case–control study of 107 patients with IBD (median age, 36.0 years; range, 22-67 years). Of the 107 patients, 53 were diagnosed with CD and 54 were diagnosed with ulcerative colitis (UC) after endoscopy and biopsy. The control group included 51 healthy subjects with a median age of 36.0 years (range, 22-69 years). CD/UC/healthy ratio was 1/1/1.

Patients were recruited during 1 year in an IBD monographic unit from the Digestive Diseases Unit of the University “Hospital Virgen Macarena” (Seville). Inclusion criteria were as follows: age between 22 and 70 years, diagnosis of IBD after endoscopy and bowel biopsy, and acquisition of informed consent. Exclusion criteria were as follows: suffering from any disease involved in bone metabolism; previous or current treatment related to bone metabolism (calcitonin, bisphosphonates, parathormone (PTH), calcium, vitamin D, anabolic steroids, androgens, hormone replacement therapy, fluor therapy) and secondary osteoporosis (rheumatoid arthritis or renal, hepatic, or endocrine disease).

Controls were randomly chosen from the blood bank donors of the HUV Macarena. They were matched by sex and age with IBD patients after ruling out pathology that alters bone metabolism. The same exclusion criteria were followed.

Data compiled from all participants included age (years) at the time of inclusion, gender, weight (kg), height (cm), body mass index (BMI), age at menopause, menstrual record, contraceptive use, calcium intake (null, moderate, or high), exposure to toxins such as alcohol (null, moderate, or high) and tobacco (null, <20 cigarettes/day, or >20 cigarettes/day), previous vertebral crush fracture, and intense or frequent back pain. In patients with IBD, age at the beginning of the disease, years of evolution, type of disease (CD or UC), corticoid treatment and duration (more or less than 1 year), bowel resection, and lactose intolerance (by the hydrogen breath test) were also included.

All participants provided written informed consent, and the study was approved by the ethics committee of this hospital.

### Measurement of dorsal and lumbar spinal X-rays

To establish the prevalence of vertebral crush fractures, posteroanterior and lateral X-rays of the dorsal and lumbar spine were performed in all participants, including the area from T4 to L4. On the lateral view, six points were considered on each vertebra to determine the anterior, middle, and posterior height of the analyzed vertebrae. The assessment for the presence of morphometric fractures was performed in a blinded fashion by two independent observers who did not know whether the radiograph was of a case or control subject. They used the semi-quantitative method (Genant’s classification [13,14] as modified by Black) [15]. A morphometric vertebral fracture was considered when a decrease of at least 20% was observed in any height measurement; compared with the others, this was a difference of >4 mm. Deformities and fractures due to nonosteoporotic causes (trauma or degenerative deformities) were not considered.

### Bone mineral density measurement

We measured bone mineral density (BMD) by dual energy X-ray absorptiometry (QDR-1000 Hologic densitometer; Waltham, MA, USA) of the lumbar spine (L2–L4) and proximal right femur. Results are expressed in absolute values (gHA/cm^2^). According to the WHO recommendations for postmenopausal women, BMD was defined as normal with a T-score of > −1 SD, osteopenia as a T-score of < −1 and > −2.5 SD, and osteoporosis as a T-score of < −2.5.

### Biochemical parameters

Blood samples were taken at baseline to determine the following serum values: calcium (mg/dl), phosphorus (mg/dl), alkaline phosphatase (U/dl), osteocalcin (ng/ml), C-reactive protein (CRP) (mg/dl), parathormone (PTH) (pg/ml), 25-hydroxycholecalciferol (25-OH-D3) (ng/ml), serum osteoprotegerin (OPG) (pg/ml), and receptor activator of nuclear factor-κß ligand (RANKL) levels (pg/ml).

Calcium, phosphorus, CRP, and alkaline phosphatase were determined by standard methods (self-analyzers).

Osteocalcin was measured using a commercial radioimmunoassay (RIA) kit (Metra Biosystems, Inc., CA, USA). The intra-assay variability (intra-assay coefficient of variation, IA-CV) was <3%, and the inter-assay variability (IE-CV) was <2.1%.

Intact PTH was determined by RIA (Allegro Antibody; Nichols Institute, San Juan Capistrano, CA, USA) (normal range, 10–65 pg/ml). The IE-CV was <3.6%, and the IA-CV was <1.2%.

Determinations of 25-OH-D3 were performed by RIA (Incstar Corporation, Stillwater, MN, USA) (normal range, 9–45 ng/ml). The IA-CV was 4.5%, and the IE-CV was 4.9%.

Serum OPG and RANKL levels were quantified by enzyme-linked immunosorbent assay (ELISA; Inmundiagnostik Bensheim and Biomedica, Austria). The detection limit was 0.14 pmol/l for OPG, and the IA-CV was 4%; for RANKL, the detection limit was 0.08 pmol/l, and the IA-CV was 4%.

Samples were stored at −80°C until measurement to avoid interassay variability.

### Statistical analysis

Quantitative variables were expressed as mean and standard deviations. Qualitative variables were compared using the chi-square test. For normal distributions, Student’s *t* test was applied. For variables without normal distributions, the Mann–Whitney *U* test was applied. Normality was verified using the Kolmogorov–Smirnov test. Correlation between variables was determined using Pearson’s correlation coefficient for normal variables and Spearman’s coefficient for variables without a normal distribution. Bivariate analysis was applied to calculate the predictive values of each variable. Analysis was statistically significant when *p* < 0.05 and the level of confidence was 95%. Beta error was set at 20%. SPSS 17.0 for Windows (SPSS, Inc., Chicago, IL, USA) was used for data analysis.

## Results

We analyzed data from 107 patients with IBD (average age, 37.4 ± 10.4 years); 53 were diagnosed with CD and 54 were diagnosed with UC after endoscopy and biopsy; 64.5% were men and 35.5% were women. In the group of women, 23.7% were menopausal (mean age, 50.5 ± 11.0 years). In the control group, we analyzed 51 healthy subjects with an average age of 39.3 ± 11.0 years, including 43.1% men and 56.9% women. Of women in the control group, 27.5% were menopausal (mean age, 52.1 ± 9.1 years).

Disease onset was earlier in patients with CD than in those with UC (median, 25.9 ± 9.2 vs. 31.5 ± 9.8 years).

Of the 53 patients with CD, 74.5% were diagnosed between 17 and 40 years of age (A2 of the Montreal classification), and 25.45% were diagnosed after age 40 (A3 of the Montreal classification). None were diagnosed at <22 years of age.

In terms of disease location, 26.82% had involvement of the terminal ileum (L1 of the Montreal classification), 34.16% had involvement of the colon (L2 of the Montreal classification), and 39.02% had involvement of the ileocolon (L3 of the Montreal classification). No patient had isolated involvement of the upper gastrointestinal tract (L4 of the Montreal classification).

In terms of the clinical pattern, 68.29% of patients had a nonstricturing and nonpenetrating pattern (B1 of the Montreal classification), 7.31% had a stricturing pattern (B2 of the Montreal classification), and 24.39% had a penetrating pattern (B3 of the Montreal classification). The B3/B3p ratio was 2.0.

Of the 54 patients with UC, according Montreal classification of the extent of UC, 34.78% had ulcerative proctitis (E1), 26.08% had involvement in the distal colon (E2), and 39.13% had pancolitis (E3). No patient had short bowel syndrome.

The extension and location of the inflammatory process had no significant influence on BMD or vertebral fractures in patients with CD nor in patients with UC.

No significant differences were observed in any of the anthropometric parameters studied between the two groups.

### Vertebral crush fractures

In our study, the prevalence of morphometric vertebral crush fractures was higher in patients with IBD than in controls of the same age and gender.

Forty-one patients with IBD (44.5%; average age, 36.7 ± 9.8 years) had a history of one or more fractures; 26 were men (average age, 38.92 ± 10.5 years), and 15 were women (average age, 33.0 ± 7.4 years). In this group, 72 fractures were finally reported. In these patients, vertebral deformities were grade I (68.29%) and II (31.7%) according to Genant’s classification. In the control group, seven patients suffered fractures (13.7%); five were men (average age, 54.0 ± 11.1 years) and two were women (average age, 51.0 ± 9.8 years). The total number of fractures was 10. In this group, vertebral deformities were grade I (57.14%), II (28.57%), or III (14.28%).

The proportion of patients with fractures was identical in patients with CD or UC (39.6% vs. 38.8%), although it was higher in both than in the control group (*p* < 0.0001). Moreover, vertebral crush fractures occurred earlier in IBD patients as seen by the difference in age (IBD, 36.75 ± 9.8 years old vs. controls, 53.41 ± 10.0 years old; *p* = 0.004).

Fractures were more frequent in men than in women in the IBD and control groups, although differences were not statistically significant.

Bone mass was not directly related to fractures in the IBD group: differences in BMD were not found between patients with or without fractures in either the lumbar spine or the femur. Notably, most patients with fractures had a normal bone mass (40.5%); 35.7% were osteopenic, and 21.4% manifested osteoporosis.

We found no correlation between fractures and BMD.

Control group subjects with vertebral crush fractures, however, were older than those without fractures (53.1 ± 10.0 vs. 37.1 ± 9.5; *p* = 0.005) in both the lumbar spine and femur. Bone mass was significantly lower in patients with fractures (Tables 1 and 2). In the fracture control group, 42.8% of patients had normal bone mass and 57.1% had osteopenia. A statistically significant negative correlation was observed between fractures and BMD in both the lumbar spine (r = −0.365; *p* = 0.01) and femur (r = −0.30; *p* = 0.02) in this group.

**Table 1:** BMD in IBD patients with or without fractures

	**BMD spine**	**BMD femoral neck**
Patients fractured (41)	0.937 ± 0.19	0.776 ± 0.17
Patients non fractured (66)	0.938 ± 0.12	0.793 ± 0.11
p	NS	NS

**Table 2:** BMD in control group with or without fractures

	**BMD spine**	**BMD femoral neck**
Controls fractured (7)	0.859 ± 0.18	0.713 ± 0.9
Controls non fractured (44)	1.030 ± 0.15	0.873 ± 0.9
p	0.004	0.007

Age and fracture were not related in the IBD group, in which 65.8% of patients with fractures were <40 years old, and a higher incidence of fracture was noted between 22 and 39 years of age. On the contrary, fractures in the control group were not present before 43 years of age.

Menopause in women with IBD did not determine the appearance of fractures or differences in bone mass or biochemical parameters between patients with IBD and control subjects.

A correlation between fracture and corticosteroid treatment was not observed. When analyzing corticosteroid treatment in fracture patients with IBD, 14.63% did not require corticosteroids, 65.85% were treated for <1 year, and 19.51% were continuously treated for periods of >1 year. In patients with fractures, 22% had never required corticosteroids, 50% were treated sporadically for <1 year, and 28% were treated for >1 year.

In both the IBD and control groups, the fractures were located between D5 and L4; 47% were located between D7 and D9.

Back pain was not associated with fracture in either patients with IBD or healthy subjects (IBD, r = 0.066, *p* = 0.53; healthy group, r = −0.117, *p* = 0.41). A high percentage of patients with fractures were asymptomatic in the IBD group (46.3%) and control group (71.4%).

In our study, patients with IBD presented a risk of fracture 4.03 times higher than that of controls (OR, 4.03; 95% CI, 1.652–9.847; *p* < 0.002). In the multivariate analysis, the only observed predictive factor related to fracture was IBD.

### BMD

Bone mass in the lumbar spine was significantly lower in patients with IBD than in controls (0.940 ± 0.15 vs. 1.008 ± 0.16 gHA/cm^2^; *p* = 0.01) and the femur (0.836 ± 0.17 vs. 0.718 ± 0.13 gHA/cm^2^; *p* = 0.04). Differences between patients with CD and those with UC were not found.

To avoid the effects of menopause, only premenopausal women were evaluated. Bone mass was also found to be significantly lower in the IBD group in the lumbar spine (0.937 ± 0.13 vs. 1.028 ± 0.13 gHA/cm^2^; *p* = 0.02). Differences in the femur were not observed.

A total of 42.05% of patients with IBD had normal bone mass, 43.92% had osteopenia, and 14.01% had osteoporosis.

In the control group, 65.3% had normal bone mass, 34.7% had osteopenia, and 0% had osteoporosis. After applying the chi-square test, statistically significant differences (*p* = 0.01) were found in the distribution of both groups.

Despite a lower bone mass with frequent corticosteroid use, statistically significant differences were not found between patients treated or not treated with corticosteroids or between patients on corticosteroids for more or less than a year, continuously or sporadically. Therefore, these parameters were not correlated. Corticosteroid use was very similar between patients with CD and those with UC.

### Biochemical parameters and bone remodeling

In patients with IBD, PTH, calcium, phosphorus, and alkaline phosphatase were within their respective normal ranges. Differences among these parameters were not found when classifying them according to type of disease (CD or UC) or when related to corticoid use.

In the IBD group, 55.14% of patients showed serum vitamin D levels of <30 ng/ml, 25 (23.3%) patients had levels of <20 ng/ml, and 5 had levels of <12 ng/ml. However, vitamin D levels were similar to those in the control population without significant differences between the populations (27.2 ± 11.4 vs. 24.03 ± 19.1; *p* = 0.06, NS). Differences in vitamin D levels between patients with or without fractures were not found.

Higher PTH serum levels were seen in patients with lactose intolerance compared with those without (42.03 ± 16.0 vs. 32.01 ± 17.6 pg/ml; *p* = 0.04).

CRP was identical in patients with or without fractures. However, CRP levels were higher in patients with IBD and bone mass levels were < −1 SD compared with patients with BMD > −1 SD (10.0 ± 11.7 vs. 5.3 ± 3.8; *p* = 0.03).

Serum osteocalcin was not different between patients with IBD and healthy subjects, regardless of whether or not they suffered fractures (3.36 ± 2.7 vs. 3.51 ± 2.4 ng/ml).

OPG and RANKL levels, although slightly higher in osteoporotic patients with IBD, were not related to bone mass, showing similar values in patients with osteopenia, osteoporosis, and normal bone mass as well as in patients with or without a history of fractures. A correlation was only found between OPG and PTH serum levels in fracture patients with IBD (r = 0.520; *p* = 0.02). This association was not found in IBD patients without fractures.

In the multivariate analysis, none of the measured parameters was significantly predictive of fracture.

## Discussion

Our data represent the first case–control study consecutively compiled in a monographic unit during a 1-year period, pairing controls and cases during the same period of time. The study included patients with CD or UC and involved assessment of BMD in all subjects, measuring the prevalence of vertebral fractures with a semiquantitative method and measuring biochemical parameters related to bone metabolism.

In this study, the proportion of morphometric vertebral fractures in patients with IBD was significantly higher (38.3%) than that in the control population (13.7%). After analyzing other publications, the prevalence of osteoporotic fractures in patients with IBD varies according to the different studies. Some authors found a low prevalence (<14%) of vertebral fractures in IBD [11,16], and others observed prevalences between 20% and 27% [3,9,10,17]. An adequate number of cases was included in some studies, although only IBD populations were assessed without comparison with control populations [3,11,16]. In other studies, the incidence of fracture in patients with IBD was estimated based on previous imaging studies without using a semiquantitative method [18]. Other studies only included patients with CD [8-11] or patients with low bone mass [8] without comparing them with a control group of healthy subjects studied in parallel. The prevalence of fractures found in the present study is higher than that in previous studies, and this could be explained by differences between the sources used for data collection and different objectives.

Differences in the prevalence of fractures between patients with CD or UC have not been observed in line with other authors [16]. Although many authors have reported a higher prevalence of vertebral fractures in patients with CD [8-11,17], there are also studies that found a higher prevalence of fractures in patients with UC [3].

Patients with IBD developed fractures at earlier ages than did controls, consistent with other publications [8-11]. Only Bernstein et al. presented conflicting data in patients <50 years of age with IBD [19].

The prevalence of vertebral fractures in our control group was somewhat lower than that in large-population studies in Europe, although in those studies, the included patients were older; thus, it is expected that the prevalence is higher [20-22].

The risk of fracture due to impact in our population was higher than that in other studies (OR, 4.03%; 95% CI, 1.652–9.847; *p* < 0.002). There are published studies that found no differences in fracture risk among patients with IBD and control populations [22,23]. Other authors have shown a low risk of fracture in patients with IBD (RR, 1.2–1.5) [24-27]. A study of a Canadian population showed an increased risk of fracture in patients with UC (RR, 1.54 vs. 1.90) [18]. These studies included a large number of patients and controls, but the information comes from questionnaires administered to patients without radiological confirmation of the fracture. In our study, the higher risk is explained by more exhaustive detection that was based on radiological study.

Like the results of most authors, our results show a significantly lower BMD in patients with IBD than in controls in both the lumbar spine and femur [3,9,17,28,29]. Differences in age or type of disease have not been found, consistent with other studies [9,10].

Back pain, a clinical expression of vertebral fracture, was not related to the presence of morphometric fracture. Back pain was present in both groups (IBD and healthy subjects) regardless of vertebral fracture. Silent fractures were observed in 58.54% of patients with IBD and in 35.0% of healthy subjects. Therefore, this clinical manifestation is not considered useful for the diagnosis of fracture [8].

Menopause has scarcely been studied in patients with IBD in relation to osteoporotic fractures. In some studies, the appearance of fractures has been found to have no relationship with menopause, which is similar to our results [17-19]. In our study, premenopausal women had a higher number of fractures than did postmenopausal women. It is noteworthy that in the group of premenopausal women with IBD, BMD already showed significantly lower values, and 12 cases of fractures were reported at an average age of 31.8 ± 5 years. Sex hormone deficiency had no significant influence on either vertebral fracture or low BMD in our patients, even as a known risk factor for low bone mass.

Corticosteroid use remains a controversial issue in the development of osteoporotic fractures in patients with IBD. The need for corticosteroid treatment in many cases of IBD has led to the hypothesis that this may be an important factor involved in the appearance of osteoporotic fractures. It is well known that corticoids exert a deleterious effect on bone, and, although contradictory results have been published in patients with IBD [10,11,16,17], some authors propose corticosteroid use as a predictive factor for osteoporotic fracture [11,17]. Others, in accordance with our results, do not consider corticosteroid use to be causal of fracture in patients with IBD [10,16,23].

Our results show a poor association between the incidence of fracture and high doses of corticoids. Oddly, in our study, those patients with higher doses and continuous corticosteroid use showed a lower number of fractures than did those only treated during exacerbation of the disease. The fact that most patients only used corticosteroids for short periods of time (exacerbation phase) may explain the less pernicious effect on bone.

The biochemical and bone remodeling parameters assessed in this study did not show significant differences in patients with IBD with or without fractures or in healthy subjects.

However, a high proportion of patients with IBD showed suboptimal levels of vitamin D (30 ng/ml) for correct bone metabolism, although the levels were not different from those in control subjects. These data have been corroborated by other studies [30-33].

Differences in CRP were not found between patients with or without fractures. The IBD group did not show inflammatory activity at the time of inclusion in this study, which justifies our results.

OPG serum levels were similar in patients with and without fractures. Several authors [34,35] justify elevated OPG levels in IBD patients with chronic bowel inflammatory activity. In our study, patients were not in an acute phase of the disease, and osteoporotic fractures did not occur at the time of inclusion. It is likely that OPG levels were not different in the studied patients when evaluating for the presence of fractures or lower bone masses because of these differences.

## Conclusions

The results of this study clearly show a higher prevalence of morphometric vertebral fractures in patients with IBD compared with a healthy population with similar characteristics (OR, 4.03). A high percentage of these fractures showed no clinical evidence. No association with BMD, age, or corticosteroid use was found because most fractures were observed in patients with normal bone mass who were younger and had lower corticosteroid use than the population with fractures. From the studied parameters, having the disease was the only factor proven to be predictive of fracture, supporting the hypothesis of the important role played by proinflammatory cytokines on vertebral fractures and low bone mass.

Lower bone mass and a high proportion of hypovitaminosis D were observed in patients with IBD. Based on these results, we consider the early detection of morphometric fracture by X-ray to be very important regardless of clinical symptoms and bone mass. Early preventive and therapeutic measures are necessary to prevent the occurrence of complications.

## Abbreviations

BMD, Bone mineral density; BMI, Body mass index; CD, Crohn’s disease; CRP, C-reactive protein; DEXA, Dual Energy X-ray Absorptiometry; gHA, Grammes of hydroxyapatite; HST, Hormone replacement therapy; IBD, Inflammatory Bowel Disease; IA-CV, Intra-assay variability coefficient; IE-CV, Inter-assay variability coefficient; L2-L4, Lumbar vertebra; MVF, Morphometric vertebral fractures; OPG, Osteoprotegerin; PTH, Parathormone; RANKL, Receptor activator for nuclear factor-κß ligand; T4, Thoracic vertebra; UC, Ulcerative colitis; 25-OH-D3, 25-hydroxycholecalciferol.

## Competing interests

None of the contributing authors has any financial or non-financial competing interests influencing interpretation of data or presentation of information.

## Authors’ contributions

All authors have contributed equally to this work. MAV: Selected patients, carried out the morphometric study and drafted the manuscript. EL: Selected patients and carried out the morphometric study. MJM: Selected patients, carried out the morphometric study and the statistical analysis. MG: Carried out the biochemical parameters and the statistical analysis. RPT: Carried out the bone mineral density measurements. RPC: Designed the study and drafted the manuscript. All authors read and approved the final manuscript.

## Pre-publication history

The pre-publication history for this paper can be accessed here:

http://www.biomedcentral.com/1471-230X/12/47/prepub
